# Monogenea of fishes from the lagoon flats of Palmyra Atoll in the Central Pacific

**DOI:** 10.3897/zookeys.713.14732

**Published:** 2017-11-02

**Authors:** Víctor Manuel Vidal-Martínez, Lilia Catherinne Soler-Jiménez, Ma. Leopoldina Aguirre-Macedo, John Mclaughlin, Alejandra G. Jaramillo, Jenny C. Shaw2, Anna James, Ryan F. Hechinger, Armand M. Kuris, Kevin D. Lafferty

**Affiliations:** 1 Laboratorio de Parasitología, Centro de Investigación y de Estudios Avanzados del IPN (CINVESTAV-IPN) Unidad Mérida, Carretera Antigua a Progreso Km. 6, Mérida, Yucatán C.P. 97310, México; 2 Department of Ecology, Evolution and Marine Biology and Marine Science Institute, University of California, Santa Barbara CA 93106, USA; 3 Scripps Institution of Oceanography-Marine Biology Research Division, University of California, San Diego, La Jolla, California 92093 USA; 4 Western Ecological Research Center, U.S. Geological Survey, Marine Science Institute, University of California, Santa Barbara CA 93106, USA

**Keywords:** Monogenea, fish, geographical isolation, islands, Indo-Pacific, atoll

## Abstract

A survey of the monogeneans of fishes from the lagoon flats of Palmyra Atoll detected 16 species already reported from the Indo-West Pacific faunal region. A total of 653 individual fish from 44 species were collected from the sand flats bordering the lagoon of the atoll. Eighteen species of fish were infected with monogeneans. The monogenean species recovered were: *Benedenia hawaiiensis* on *Acanthurus xanthopterus*, *Chaetodon auriga*, *Chaetodon lunula*, *Mulloidichthys flavolineatus*, *Pseudobalistes flavimarginatus* and *Rhinecanthus aculeatus*; *Ancyrocephalus ornatus* on *Arothron hispidus*; *Euryhaliotrema annulocirrus* on *Chaetodon auriga* and *Chaetodon lunula*; *Euryhaliotrema chrysotaeniae* on *Lutjanus fulvus*; *Euryhaliotrema grandis* on *Chaetodon auriga* and *Chaetodon lunula*; *Haliotrema acanthuri* on *Acanthurus triostegus*; *Haliotrema aurigae* on *Chaetodon auriga* and *Chaetodon lunula*; *Haliotrema dempsteri* on *Acanthurus xanthopterus*; *Haliotrema minutospirale* on *Mulloidichthys flavolineatus*; *Haliotrematoides patellacirrus* on *Lutjanus monostigma*; *Neohaliotrema bombini* on *Abudefduf septemfasciatus* and *Abudefduf sordidus*; *Acleotrema girellae* and *Acleotrema parastromatei* on *Kyphosus cinerascens*; *Cemocotylella elongata* on *Caranx ignobilis*, *Caranx melampygus* and *Caranx papuensis*; *Metamicrocotyla macracantha* on *Crenimugil crenilabris*; and *Pseudopterinotrema albulae* on *Albula glossodonta*. All these monogenean–host combinations represent new geographical records. The monogenean species composition of the Palmyra Atoll is similar to that of the Hawaiian Islands. However, the number of species recovered was lower compared with other localities within the Indo-West Pacific, perhaps due to the geographical isolation of Palmyra Atoll.

## Introduction

Several studies on the parasitic fauna of marine fishes have been conducted at different localities in the Indo-West Pacific, including off the Great Barrier Reef (Australia), New Caledonia, Moorea (French Polynesia), Malaysia, South China, and the Hawaiian Islands (Yamaguti 1965, 1968, Young 1968, Plaisance et al. 2004, Plaisance and Kritsky 2004, Lim and Justine 2007, Kritsky et al. 2009, Lim and Gibson 2008, 2009, 2010, Rehulkova et al. 2010, Palm and Bray 2014, Mendoza-Franco et al. 2017).

Palmyra Atoll is one of the northern Line Islands located in the East Indo-Pacific marine ecoregion (Spalding et al. 2007), 1680 km south-south-west of Hawaii. It is presently a marine protected area and lacks regular human settlement since World War II. The Palmyra Atoll represents a relatively long history with little to no exploitation (Lafferty et al. 2008). All fishing has been prohibited at Palmyra since it became a US National Wildlife Refuge in 2000 (before that, its remoteness kept fishing pressure low). As a part of a larger research project on the role of infectious agents in the Palmyra Atoll ecosystem, we had the opportunity to undertake a survey of the fish parasites from the lagoon flats of this coral atoll. The goals of the present paper were to report the monogenean species recovered and to establish their zoogeographical affinities with respect to the Indo-West Pacific (IWP) ecoregion.

## Methods

Between 13 October and 10 November 2009, and 22 June and 28 July 2010, we captured fishes by seine, spear, and hook and line from the intertidal sand flats bordering the lagoon of Palmyra Atoll (5°53′00″N; 162°05′00″W), U.S.A. Immediately after capture, the fish were separated and anesthetised individually with 0.5 ml L^−1^ of 2-phenoxyethanol (Sigma, St. Louis, MO, USA) in plastic bags with lagoon water to avoid loss or mixing of monogeneans among fishes and transported them to the laboratory facility of the Palmyra Atoll Research Consortium (PARC). We examined only freshly killed fish (and the bag water). Observations were under a stereomicroscope with a total magnification of 40×. For each individual host, the skin was examined and the gill arches removed, examined, and the monogeneans obtained were counted, preliminarily identified, and most of them (70–80%) fixed in 4% hot formalin, labelled, and stored in vials for later evaluation. The remaining specimens were flattened and mounted in glycerine ammonium picrate mixture (GAP) to study the morphology of sclerotized structures under a compound microscope (Olympus BX-53, Olympus Corporation, Tokyo, Japan). After evaluation, the specimens that had been fixed with GAP were remounted in Canada balsam (Ergens 1969). Unflattened specimens were cleared with Gray & Wess medium or stained in trichrome and mounted in Canada balsam (for details of these techniques, see Vidal-Martínez et al. 2001). In this manuscript, the male copulatory organ of the monogeneans is denoted below as the MCO. Prevalence and mean intensity concepts were applied following Bush et al. (1997). Specimen of each species were deposited in the United States National Parasite Collection, NMNH Invertebrate Zoology, Smithsonian Museum Support Center, MD, USA (USNM), and the Helminthological Collection of the Laboratory of Parasitology, at Centre for Research and Advanced Studies, National Polytechnic Institute, Mérida, Yucatán, México (CHCM).

## Results

### Monogeneans of fishes from the Palmyra lagoon flats

During this study, 653 individual fish belonging to 44 species were collected (Table 1). The 16 species of monogeneans infected 18 fish species (Table 2). Those hosts with the most monogenean species were *Chaetodon auriga* Forsskål and *Chaetodon lunula* (Lacépède) with four species, and *Kyphosus cinerascens* (Forsskål), *Acanthurus xanthopterus* Valenciennes and *Mulloidichthys flavolineatus* (Lacépède) with two species each. All other fish species harboured one monogenean species. Twenty-six additional fish species were examined, but no monogeneans were found (Table 2).

**Table 1.** Fish species examined from the lagoon flats of Palmyra Atoll. N = number of fish examined; Max = maximum length reported for that fish species in FishBase (http://www.fishbase.se); Range = total length range of the fish examined.

Host examined	Fish common name	N	Infected hosts	Max (cm)	Range (cm)
**Acanthuridae**					
*Acanthurus triostegus* (Linnaeus, 1758)	Convict surgeon	50	22	27	10–18
*Acanthurus xanthopterus* Valenciennes, 1835	Yellowfin surgeon	20	16	70	20–40
**Albulidae**					
*Albula glossodonta* (Forsskål, 1775)	Roundjaw bonefish	24	17	90	37–58
**Apogonidae**					
*Cheilodipterus quinquelineatus* Cuvier, 1828	Five-lined cardinalfish	5	0	13	5–6
**Balistidae**					
*Pseudobalistes flavimarginatus* (Rüppell, 1829)	Yellowmargin triggerfish	4	1	60	17–53
*Rhinecanthus aculeatus* (Linnaeus, 1758)	Blackbar triggerfish	18	3	30	8–24
**Belonidae**					
*Platybelone argalus* (Lesueur, 1821)	Keeltail needlefish	2	0	50	9–36
**Carangidae**					
*Carangoides ferdau* (Forsskål, 1775)	Blue trevally	5	0	75	33 – 38
*Carangoides orthogrammus* (Jordan & Gilbert, 1882)	Island trevally	3	0	75	25 – 35
*Caranx ignobilis* (Forsskål, 1775)	Giant trevally	4	2	170	56–79
*Caranx melampygus* Cuvier, 1833	Bluefin trevally	6	1	117	31–66
*Caranx papuensis* Alleyne & MacLeay, 1877	Brassy trevally	5	2	88	12–41
**Carcharhinidae**					
*Carcharhinus melanopterus* (Quoy & Gaimard, 1824)	Blacktip reef shark	5	0	200	46–219
**Chaetodontidae**					
*Chaetodon auriga* Forsskål, 1775	Threadfin butterflyfish	13	9	23	12–19
*Chaetodon lunula* (Lacépède, 1802)	Raccoon butterflyfish	14	11	20	11–16
**Chanidae**					
*Chanos chanos* (Forsskål, 1775)	Milkfish	5	0	180	31–57
**Gobiidae**					
*Amblygobius phalaena* (Valenciennes, 1837)	Whitebarred goby	18	0	15	1.3–7
*Asterropteryx semipunctata* Rüppell, 1830	Starry goby	12	0	6	2–4
*Gnatholepis anjerensis* (Bleeker, 1851)	Eye-bar goby	2	0	8	2–3
*Istigobius decoratus* (Herre, 1927)	Decorated goby	5	0	13	7–11
*Istigobius ornatus* (Rüppell, 1830)	Ornate goby	26	0	11	3–6
*Istigobius rigilius* (Herre, 1953)	Rigilius goby	1	0	11	4
*Oplopomus oplopomus* (Valenciennes, 1837)	Spinecheek goby	26	0	10	2–7
*Psilogobius prolatus* Watson & Lachner, 1985	Longjaw shrimpgoby	11	0	6	2–4
*Valenciennea sexguttata* (Valenciennes, 1837)	Sixspot goby	14	0	14	2–9
**Hemiramphidae**					
*Hemiramphus depauperatus* Lay & Bennett, 1839	Tropical half-beak fish	20	0	40	20–34
**Kyphosidae**					
*Kyphosus cinerascens* (Forsskål, 1775)	Blue sea chub	2	2	50	35–38
**Lutjanidae**					
*Lutjanus fulvus* (Forster, 1801)	Blacktail snapper	26	13	40	7–26
*Lutjanus monostigma* (Cuvier, 1828)	One spot snapper	6	2	60	17–37
**Mugilidae**					
*Crenimugil crenilabis* (Forsskål, 1775)	Fringelip mullet	42	2	60	8–45
*Liza vaigiensis* (Quoy & Gaimard, 1825)	Squaretail mullet	54	0	63	3–32
*Valamugil engeli* (Bleeker, 1858)	Kanda	63	0	30	1–20
**Mullidae**					
*Mulloidichthys flavolineatus* (Lacépède, 1801)	Yellowstripe goatfish	52	10	43	8–37
*Upeneus taeniopterus* Cuvier, 1829	Finstripe goatfish	5	0	33	1–30
**Muraenidae**					
*Gymnothorax pictus* (Ahl, 1789)	Paintspotted moray	7	0	140	41–70
**Ophichthidae**					
*Myrichthys colubrinus* (Boddaert, 1781)	Harlequin snake eel	3	0	97	33–65
**Pinguipedidae**					
*Parapercis lata* Randall & McCosker, 2002	Y-Barred Sandperch	13	0	21	2–3
**Pomacentridae**					
*Abudefduf septemfasciatus* (Cuvier, 1830)	Banded sergeant	12	3	23	14–20
*Abudefduf sordidus* (Forsskål, 1775)	Blackspot sergeant	18	5	24	14–19
*Chrysiptera glauca* (Cuvier, 1830)	Grey demoiselle	3	0	12	8–10
*Stegastes nigricans* (Lacépède, 1802)	Dusky farmerfish	10	0	14	8–10
**Serranidae**					
*Epinephelus merra* Bloch, 1793	Honeycomb grouper	2	0	32	13–24
**Sphyraenidae**					
*Sphyraena barracuda* (Edwards, 1771)	Great barracuda	2	0	200	65–76
**Tetraodontidae**					
*Arothron hispidus* (Linnaeus, 1758)	White–spotted puffer	15	14	50	17–49

**Table 2.** Monogeneans of fishes from the lagoon flats of Palmyra Atoll; N = number of fish examined.

	Hosts	N	Infected hosts	Prevalence (%)	Mean intensity (± SD)
**Capsalidae**					
*Benedenia hawaiiensis*	*Acanthurus xanthopterus*	20	1	5	2
	*Chaetodon auriga*	13	1	7,7	1
	*Chaetodon lunula*	14	2	14,3	1 ± 0
	*Mulloidichthys flavolineatus*	52	4	7,7	2 ± 0,6
	*Pseudobalistes flavimarginatus*	4	1	25	1
	*Rhinecanthus aculeatus*	18	3	16,7	3 ± 2
**Dactylogyridae**					
*Ancyrocephalus ornatus*	*Arothron hispidus*	15	14	93,3	47 ± 69
*Euryhaliotrema annulocirrus*	*Chaetodon auriga*	13	7	53,8	22 ± 22
	*Chaetodon lunula*	14	10	71,4	42 ± 18
*Euryhaliotrema chrysotaeniae*	*Lutjanus fulvus*	26	13	50	15 ± 22
*Euryhaliotrema grandis*	*Chaetodon auriga*	13	4	30,8	17 ± 19
	*Chaetodon lunula*	14	5	35,7	28 ± 19
*Haliotrema acanthuri*	*Acanthurus triostegus*	50	22	44	13 ± 17
*Haliotrema aurigae*	*Chaetodon auriga*	13	4	30,8	61 ± 49
	*Chaetodon lunula*	14	5	35,7	66 ± 20
*Haliotrema dempsteri*	*Acanthurus xanthopterus*	20	16	80	35 ± 28
*Haliotrema minutospirale*	*Mulloidichthys flavolineatus*	52	10	19,2	27 ± 18
*Haliotrematoides patellacirrus*	*Lutjanus monostigma*	6	2	33,3	145 ± 197
*Neohaliotrema bombini*	*Abudefduf septemfasciatus*	12	3	25	4 ± 2
	*Abudefduf sordidus*	18	5	27,8	138 ± 97
**Diplectanidae**					
*Acleotrema girellae*	*Kyphosus cinerascens*	2	2	100	84 ± 90
*Acleotrema parastromatei*	*Kyphosus cinerascens*	2	1	50	50
**Heteraxinidae**					
*Cemocotyllela elongata*	*Caranx ignobilis*	4	2	50	7 ± 7
	*Caranx melampygus*	6	1	16,7	4
	*Caranx papuensis*	5	2	40	7 ± 7
**Microcotylidae**					
*Metamicrocotyla macracantha*	*Crenimugil crenilabis*	42	2	4,8	3 ± 1
**Pterinotrematidae**					
*Pseudopterinotrema albulae*	*Albula glossodonta*	24	17	70,8	17 ± 18

### Monogenea van Beneden, 1858

#### Monopisthocotylea Odhner, 1912

##### Capsalidae Baird, 1853

###### Benedenia hawaiiensis

Taxon classificationAnimaliaCapsalideaCapsalidae

Yamaguti, 1968

####### Type host.

*Priacanthus cruentatus* (Lacépède) (Priacanthidae)

####### Other host and localities.

*Benedenia hawaiiensis* has been reported from more than 24 species of fishes from off Hawai’i (Whittington et al. 2001). From *Sargocentron spiniferum* (Forsskål) (Holocentridae) in the South China Sea (as *Benedenia sargocentron*) (Zhang et al. 2001).

####### Current host.

*Acanthurus xanthopterus* (Acanthuridae), *Chaetodon auriga*, *Chaetodon lunula* (Chaetodontidae), *Mulloidichthys flavolineatus* (Lacépède) (Mullidae), *Pseudobalistes flavimarginatus* (Rüppell) and *Rhinecanthus aculeatus* (Linnaeus) (Balistidae).

####### Site infection.

Gills.

####### Prevalence and mean intensity.

*Acanthurus xanthopterus* 5 and 2 (n = 20); *Chaetodon auriga* 7,7 and 1 (n = 13); *Chaetodon lunula* 14,3 and 1±0 (n = 14); *Mulloidichthys flavolineatus* 7,7 and 2±0,6 (n = 52); *Pseudobalistes flavimarginatus* 25 and 1 (n = 4) and *Rhinecanthus aculeatus* 16,7 and 3±2 (n = 18).

####### Specimens deposited.

CHCM No. 551 (paratypes) (1 slide, 1 specimen).

####### Remarks.

*Benedenia hawaiiensis* was originally described by Yamaguti (1968) from the gills and fins of *Priacanthus cruentatus* off Hawai’i. Zhang et al. (2001) described *Benedenia sargocentron* on *Sargocentron spiniferum* from the South China Sea. However, Deveney and Whittington (2010) determined that *B. sargocentron* was a junior synonym of *B. hawaiiensis*, and proposed keeping *B. hawaiiensis* as the valid name. *Benedenia hawaiiensis* is characterized by having an opisthaptor which is usually a little longer than wide, provided with a marginal membrane and is notched opposite the marginal hooklets; with 14 marginal hooklets; two between the posterior anchors. The marginal valve is clearly indented at each hooklet and also at the position where the posterior anchor meets the haptor edge. The marginal valve is conspicuous and has one lobe between each of the hooklets around the circumference of the haptor with the anterior lobes being larger and wider. The proximal ends of the anterior anchors overlap the proximal ends of the accessory sclerites, and the distal portion of the anterior anchors overlap the posterior anchors for two-thirds of their lengths. The accessory sclerites are alate and raise the ventral haptoral tissues through which they protrude. The MCO is muscular, well-equipped with circular and longitudinal muscle fibres and lies in a cavity or canal with poorly developed muscle walls. The most prominent feature of the MCO of *B. hawaiiensis* is that it tapers to form a narrow distal tip. The presence of *B. hawaiiensis* on the gills of *R. aculeatus* at Palmyra Atoll represents both a new host and a new geographical record for this species.

##### Dactylogyridae Bychowsky, 1933

###### Ancyrocephalus ornatus

Taxon classificationAnimaliaDactylogyrideaDactylogyridae

Yamaguti, 1968

####### Type host.

*Arothron hispidus* (Linnaeus) (Tetraodontidae).

####### Other host and localities.

*Arothron hispidus*, Hawai’i (Yamaguti 1968).

####### Current host.

*Arothron hispidus*.

####### Site infection.

Gills.

####### Prevalence and mean intensity.

93,3 and 47 ± 69 (n=15).

####### Specimens deposited.

CHCM No. 550 (paratypes) (1 slide, 1 specimen), USNM No. 1459841 (voucher) (1 slide, 1 specimen).

####### Remarks.

Originally described by Yamaguti (1968) from gills of *A. hispidus* off Hawai’i, this species has recently been reported by Palm and Bray (2014) from the same host and locality. It is characterized by the cirrus being ornamented distally with a spiral fold, hence the specific name. New geographical record for Palmyra Atoll.

###### Euryhaliotrema annulocirrus

Taxon classificationAnimaliaDactylogyrideaDactylogyridae

(Yamaguti, 1968) Kritsky, 2012

####### Type host.

*Chaetodon auriga* (Chaetodontidae).

####### Other host and localities.

*Chaetodon auriga*, Hawai’i (Yamaguti 1968; Mizelle and Kritsky 1969), *Chaetodon lunula*, Hawai’i (Yamaguti 1968; Mizelle and Kritsky 1969), *Chaetodon bellamaris* (= *C. wiebeli*) Kaup from the South China Sea (Zhang et al. 2003), *Chaetodon modestus* Temminck and Schlegel from the South China Sea (Zhang et al. 2003), *Roa modesta* and *Chaetodon vagabundus* Linnaeus from Moorea, French Polynesia, Great Barrier Reef, Australia, Palau, New Caledonia (all Chaetodontidae).

####### Current host.

*Chaetodon auriga* and *Chaetodon lunula* (Chaetodontidae).

####### Site infection.

Gills.

####### Prevalence and mean intensity.

53,8 and 22±22 (n=13) to *C. auriga*; 71,4 and 42±18 (n=14) to *C. lunula*.

####### Specimens deposited.

CHCM No. 542 (paratypes) (1 slide, 1 specimen) (for *C. auriga*), CHCM No. 543 (paratypes) (1 slide, 2 specimen) (for *C. lunula*), USNM No. 1459842 (voucher) (1 slide, 1 specimen) (for *C. lunula*).

####### Remarks.

This species was originally described by Yamaguti (1968) as *Haliotrema annulocirrus* and transferred to the genus *Euryhaliotrematoides*, as *E. annulocirrus*, by Plaisance and Kritsky (2004). Recently, Kritsky (2012) proposed the synonymy of *Euryhaliotrematoides* with *Euryhaliotrema*. As result, this species was transferred as *Euryhaliotrema annulocirrus*. *Haliotrema annulocirrus* Yamaguti 1968, *Euryhaliotrematoides annulocirrus* (Yamaguti 1968) Plaisance and Kritsky 2004, *Parahaliotrema affinis* Mizelle and Kritsky 1969, *Haliotrema affinis* (Mizelle and Kritsky 1969) Vala et al. 1982 and *Haliotrema angulocirrus* Yamaguti 1968 are considered synonyms of this species. *Euryhaliotrema annulocirrus* is distinguished from all other species of the genus by having an enlarged slit-like vaginal pore with serrated posterior lip and a conspicuous, elongate and heavy coiled tube of the MCO. New geographical record for Palmyra Atoll.

###### Euryhaliotrema chrysotaeniae

Taxon classificationAnimaliaDactylogyrideaDactylogyridae

(Young, 1968) Kritsky & Boeger, 2002

####### Type host.

*Lutjanus chrysotaenia* (=*L. carponotatus*) (Richardson) (Lutjanidae).

####### Other host and localities.

Gills of *L. chrysotaenia* (=*L. carponotatus*) from Heron Island, Queensland, Australia (as *Haliotrema chrysotaeniae*) (Young 1968). *Lutjanus fulvus* (Forster), *Lutjanus fulviflamma* (Forsskål), *Lutjanus quinquelineatus* (Bloch) and *Lutjanus russellii* (Bleeker) from Nouméa, New Caledonia (Kritsky 2012). *Lutjanus fulvus* and *Lutjanus kasmira* (Forsskål) from French Polynesia and Hawaiian Islands (all as *Euryhaliotrema chrysotaeniae*) (Vignon et al. 2009). *Lutjanus russellii* from Guangdong Province, China (as *Euryhaliotrema chrysotaeniae*) (Li and Yan 2007) (all Lutjanidae).

####### Current host.

*Lutjanus fulvus* (Lutjanidae).

####### Site infection.

Gills.

####### Prevalence and mean intensity.

50 and 15 ± 25 (n=26).

####### Specimens deposited.

CHCM No. 537 (paratypes) (1 slide, 1 specimen).

####### Remarks.

Originally described by Young (1968) as *Haliotrema chrysotaeniae* and transferred to the genus *Euryhaliotrema* by Kritsky and Boeger (2002) as *E. chrysotaeniae*. *Euryhaliotrema chrysotaeniae* has a delicate MCO with more than three rings and an elongate meandering vaginal canal. New geographical record for Palmyra Atoll.

###### Euryhaliotrema grandis

Taxon classificationAnimaliaDactylogyrideaDactylogyridae

(Mizelle & Kritsky, 1969) Kritsky, 2012

####### Type host.

*Chaetodon auriga* (Chaetodontidae)

####### Other host and localities.

Gills of several species of Chaetodontidae. *Chaetodon auriga* and *C. lunula* in Hawai’i (as *Parahaliotrema grandis*) (Mizelle and Kritsky 1969); *Chaetodon chrysurus* (=*C. paucifasciatus*) Ahl in the Red Sea (as *Haliotrema grandis*) (Paperna 1972). Plaisance and Kritsky (2004) recorded *E. grandis* on gills of *Chaetodon auriga* and *Chaetodon citrinellus* Cuvier from the coral reefs of Micronesia, French Polynesia, Wallis, Australia and New Caledonia; *Chaetodon ephippium* Cuvier from Wallis and Lizard Island, Australia; *Chaetodon kleinii* Bloch from off Wallis, Australia and Micronesia; *Chaetodon lineolatus* Cuvier from off Heron Island, Australia; *C. lunula* from off French Polynesia, Wallis and Micronesia; *Chaetodon ornatissimus* Cuvier from French Polynesia and Wallis; *Chaetodon trifasciatus* Park from Wallis; *C. vagabundus* from the coral reefs of Micronesia, French Polynesia, Wallis, Australia and New Caledonia; and *Heniochus chrysostomus* Cuvier from off Moorea, French Polynesia. Only one chaetodontid host was found parasitised at Palmyra Atoll.

####### Current host.

*Chaetodon auriga and Chaetodon lunula.*

####### Site infection.

Gills.

####### Prevalence and mean intensity.

*Chaetodon auriga* 30.8 and 17±19 (n = 13).

and *Chaetodon lunula* 35.7 and 28±19 (n = 14).

####### Specimens deposited.

CHCM No. 544 (paratypes) (1 slide, 1 specimen), USNM No. 1459843 (voucher) (1 slide, 2 specimen).

####### Remarks.

*Euryhaliotrema grandis* was described by Mizelle and Kritsky (1969) as *Parahaliotrema grandis* and subsequently transferred to *Haliotrema* by Paperna (1972) as *Haliotrema grandis*. Plaisance and Kritsky (2004) erected the genus *Euryhaliotrematoides* and transferred several species of *Haliotrema* to this genus, including *Euryhaliotrematoides grandis*. Recently, Kritsky (2012) proposed the synonymy of *Euryhaliotrematoides* with *Euryhaliotrema*. As a consequence, *Euryhaliotrematoides grandis* was transferred to *Euryhaliotrema* as *Euryhaliotrema grandis*. *Euryhaliotrema grandis* presents a delicate MCO, comprising approximately two rings. Accessory piece variable, serving as a guide for the distal portion of the MCO and is articulated to the base of MCO. Anchors lacking hinged bases. Ventral anchor with flattened base supporting a short deep root, moderately long superficial root, short shaft and point extending to the level of the tip of the superficial root. Dorsal anchor with a short deep root, elongate superficial root, broad base, short shaft, point extending to the level of the superficial root. Ventral bar V-shaped, with an anteromedial concavity having a straight anterior margin and a posterior rounded expansion. Dorsal bar, rod shaped, straight. New geographical record for Palmyra Atoll.

###### Haliotrema acanthuri

Taxon classificationAnimaliaDactylogyrideaDactylogyridae

Yamaguti, 1968

####### Type host.

*Acanthurus sandvicensis* (=*A. triostegus*) (Linnaeus) (Acanthuridae).

####### Other host and localities.

Yamaguti (1968) recorded *Haliotrema acanthuri* from *Acanthurus sandvicensis* (=*A. triostegus*) in Hawai’i. It has also been found by Palm and Bray (2014) on *A. triostegus* also in Hawai’i (all Acanthuridae).

####### Current host.

*Acanthurus triostegus* (Acanthuridae).

####### Site infection.

Gills.

####### Prevalence and mean intensity.

44 and 13± 17 (n=50).

####### Specimens deposited.

CHCM No. 548 (paratypes) (1 slide, 1 specimen), USNM No. 1459844 (voucher) (1 slide, 1 specimen).

####### Remarks.

This species is characterized by the morphology of its copulatory complex, which has a bell-shaped base and a short cylindrical shaft, from which arises a proper MCO, and a similar, solid, shorter spike projecting from the genital pore. New geographical record for Palmyra Atoll.

###### Haliotrema aurigae

Taxon classificationAnimaliaORDOFAMILIA

(Yamaguti, 1968) Plaisance, Bouamer & Morand, 2004

####### Type host.

*Chaetodon auriga* ().

####### Other host and localities.

*Chaetodon auriga* from Hawai’i (Yamaguti 1968). On *C. auriga*, *C. citrinellus*, *C. vagabundus*, *C. ephippium*, *C. lunulatus*, *C. kleinii*, *C. lunula*, *C. ornatissimus*, *C. reticulatus*, *C. trifascialis* and *H. chrysostomus* from several sites of the Indo-West Pacific Ocean (Palau Micronesia, Moorea French Polynesia, Wallis and Futuna, New Caledonia, Heron Island and Lizard Island, Australia) (Plaisance et al. 2004). Recently, this species was reported on *C. auriga* from off the Pratas Islands, South China Sea (Kritsky et al. 2009) and Hawai’i (Palm and Bray 2014) (all ).

####### Current host.

*Chaetodon auriga* and *Chaetodon lunula* ().

####### Site infection.

Gills.

####### Prevalence and mean intensity.

30,8 and 61±49 (n=13) to *C. auriga* and 35,7 and 66±20 (n=14) to *C. lunula*.

**Specimens deposited**: CHCM No. 545 (paratypes) (1 slide, 3 specimens), USNM No. 1459845 (voucher) (1 slide, 6 specimen).

####### Remarks.

This species was described for the first time by Yamaguti (1968) as *Pseudohaliotrematoides aurigae*. In 2004, Plaisance et al. recorded this species from 10 species of *Chaetodon* and one species of *Heniochus* (Chaetodontidae), and transferred it to the genus *Haliotrema* as *H. aurigae*. *Haliotrema aurigae* presents a tubular MCO bent near base base; base trapezoid; filamentous accessory piece, elongated, serving as a guide for the distal portion of the MCO. Dorsal anchor base/shaft junction hinged, with elongate superficial root and short, deep root. Ventral anchor with short roots and broad, slightly fenestrated base. Dorsal bar straight, bone-shaped. Ventral bar rod-shaped, an inverted broad U. New geographical record for Palmyra Atoll.

###### Haliotrema dempsteri

Taxon classificationAnimaliaDactylogyrideaDactylogyridae

(Mizelle & Price, 1964) Young, 1968

####### Type host.

*Acanthurus xanthopterus*.

####### Other host and localities.

*Acanthurus mata* Cuvier, *Acanthurus dussumieri* Valenciennes and *A. xanthopterus* in Australia (Young 1968). Mizelle and Price (1964) recorded it previously from the gills of the *Zanclus canescens* (=*Z. cornutus*) (Linnaeus) (as *Parahaliotrema dempsteri*).

####### Current host.

*Acanthurus xanthopterus*.

####### Site infection.

Gills.

####### Prevalence and mean intensity.

80 and 35±28 (n=20).

####### Specimens deposited.

CHCM No. 549 (paratypes) (1 slide, 4 specimen), USNM No. 1459846 (voucher) (1 slide, 1 specimen).

####### Remarks.

*Haliotrema dempsteri* was originally described as *Parahaliotrema dempsteri* by Mizelle and Price (1964). Later, Young (1968) recorded it from *A. mata*, *A. dussumieri* and *A. xanthopterus*, redescribing and transferring it to *Haliotrema*. The most relevant morphological characteristics are: haptor subhexagonal, broader than long; one dorsal and one ventral pair of anchors, similar in size and shape; superficial root of each anchor base longer than the deep root; shafts solid and points without formation of a definite angle; wings low and inconspicuous on dorsal anchor shafts, apparently absent on ventral shafts; copulatory complex composed of an MCO and an elongate accessory piece attached to the proximal portion of the MCO shaft, terminating in a recurved tip; and MCO tubular with relatively large base and an undulate shaft. New geographical record for Palmyra Atoll.

###### Haliotrema minutospirale

Taxon classificationAnimaliaDactylogyrideaDactylogyridae

Yamaguti, 1968

####### Type host.

*Parupeneus cyclostomus* (Lacépède) (Mullidae).

####### Other host and localities.

Yamaguti (1968) recorded this species from gills of *P. cyclostomus, P. pleurostigma* (Bennett) and *P. multifaciatus* (Quoy and Gaimard) for Hawai’i. Palm and Bray (2014) also recorded this species from *P. cyclostomus* in the same locality (all Mullidae).

####### Current host.

*Mulloidichthys flavolineatus* (Mullidae) (New host).

####### Site infection.

Gills.

####### Prevalence and mean intensity.

19,2 and 27±18 (n=52).

####### Specimens deposited.

CHCM No. 539 (paratypes) (1 slide, 1 specimen), USNM No. 1459850 (voucher) (1 slide, 1 specimen)

####### Remarks.

The morphology of the copulatory complex is a relevant characteristic for its identification. Its MCO consists of an anterior, spiral, flanged portion and a posterior, cylindrical portion, enclosed in a sheath of circular muscular fibres. The presence of *H. minutospirale* on the gills *Mulloidichthys flavolineatus* from Palmyra Atoll represents both a new host and a new geographical record.

###### Haliotrematoides patellacirrus

Taxon classificationAnimaliaDactylogyrideaDactylogyridae

(Bychowsky & Nagibina, 1971) Kritsky, Yang & Sun, 2009

####### Type host.

*Lutjanus lutjanus* (Bloch) (Lutjanidae).

####### Other host and localities.

Previous records (as *Haliotrema patellacirrus*) on *L. lutjanus* and *L. fulviflamma* from South China Sea (Bychowsky and Nagibina 1971). Kritsky et al. (2009) recorded this species (as *Haliotrematoides patellacirrus*) on *L. russellii*, *L. fulvus*, *Lutjanus vitta* (Quoy and Gaimard) and *L. quinquelineatus* from off New Caledonia; *L. fulviflamma* from Australia; and *Lutjanus ehrenbergii* (Peters) from Nabq Bay, Ras Mohammed National Park (South Sinai, Red Sea) Egypt (all Lutjanidae).

####### Current host.

*Lutjanus monostigma* (Lutjanidae) (New host).

####### Site infection.

Gills.

####### Prevalence and mean intensity.

33,3 and 145±197 (n=6).

####### Specimens deposited.

CHCM No. 538 (paratypes) (1 slide, 3 specimen).

####### Remarks.

*Haliotrematoides patellacirrus* presents an MCO comprising a proximal platter-shaped base, distal tubular shaft with aloose clockwise coil of about 3/4 of a ring, enclosed in a sheath with a subterminal knob-like projection. Ventral anchor with elongate superficial root, knob-like deep root, shaft slightly narrowed distally and straight, recurved point with delicate superficial grooves. Dorsal anchors with elongate superficial root, inconspicuous (or absent) deep root, straight shaft of varying diameter and recurved point; distal shaft and point superficially grooved. Ventral bar with two submedial pockets along anterior margin; dorsal bar rod-shaped, with subterminal notches, ending slightly narrower than medial portion of bar. The presence of *H. patellacirrus* from *L. monostigma* off Palmyra Atoll represents both a new host and a new geographical record. Only one host was found parasitised.

###### Neohaliotrema bombini

Taxon classificationAnimaliaDactylogyrideaDactylogyridae

Lim & Gibson, 2010

####### Type host.

*Abudefduf vaigiensis* (Quoy and Gaimard) (Pomacentridae).

####### Other host and localities.

*Abudefduf vaigiensis* from Pulau Langkawi, Malaysia (Lim and Gibson 2010) (Pomacentridae).

####### Current host.

*Abudefduf septemfasciatus* (Cuvier) (New host) and *Abudefduf sordidus* (Forsskål) (Pomacentridae) (New host).

####### Site infection.

Gills.

####### Specimens deposited.

CHCM No. 546 (paratypes) (1 slide, 1 specimen) (for *A. septemfasciatus*), CHCM No. 547 (paratypes) (1 slide, 1 specimen) (for *A. sordidus*), USNM No. 1459851 (voucher) (1 slide, 1 specimen) (for *A. sordidus*).

####### Prevalence and mean intensity.

25 and 4±2 (n=12) to *A. septemfasciatus* and 27,8 and 138±97 (n = 18) to *A. sordidus*.

####### Remarks.

This species can be distinguished from other members in the genus by having an inconspicuously sclerotised MCO, consisting of a simple, curved, short tube with a large initial part and simple, bifid stick-like accessory piece. This species also has V-shaped bars with processes, ‘marginal’ hooks of different sizes, anchors with a spatulate, recurved and grooved point, and a non-fenestrated haptor. The presence of *N. bombini* in *A. septemfasciatus* and *A. sordidus* off Palmyra Atoll represents both new hosts and geographical records for this species.

##### Diplectanidae Monticelli, 1903

###### Acleotrema girellae

Taxon classificationAnimaliaDactylogyrideaDiplectanidae

Johnston & Tiegs, 1922

####### Type host.

*Girella tricuspidata* (Quoy and Gaimard) (Kyphosidae).

####### Other host and localities.

*Girella tricuspidata* from off Caloundra, southeast of Queensland, Australia (Johnston and Tiegs 1922). *Kyphosus cinerascens* collected off Hawai’i (as *Acleotrema kyphosi*) (Yamaguti 1968). *Kyphosus elegans* (Peters) from Chamela Bay, Mexico (as *Heteroplectanum kyphosi*) (León-Régagnon et al. 1997). *Kyphosus* spp. (as *A. girellae*) from Australia, the Mediterranean Sea and Mexican Pacific (Domingues and Boeger 2007) (all Kyphosidae).

####### Current host.

*Kyphosus cinerascens* (Kyphosidae).

####### Site infection.

Gills.

####### Specimens deposited.

CHCM No. 540 (paratypes) (1 slide, 2 specimen), USNM No. 1459852 (voucher).

####### Prevalence and mean intensity.

100 and 84±90 (n=2).

####### Remarks.

*Acleotrema girellae* was originally described from the gills of *G. tricuspidata* collected off Caloundra, southeast Queensland, Australia (Johnston and Tiegs 1922). In 1937, Price transferred this species to *Diplectanum* as *D. girellae*, considering *Acleotrema* a junior synonym of *Diplectanum*, based on the presence of squamodiscs. However, Yamaguti (1963) accepted *Acleotrema* as a valid genus. Rakotofiringa et al. (1987) proposed the genus *Heteroplectanum* and several species have been transferred to this new genus, including *Diplectanum kyphosi* (considered a synonym of *A. girellae*) as *Heteroplectanum kyphosi* (Yamaguti, 1968) Oliver 1987. However, Domingues and Boeger (2007) considered that species of *Acleotrema* share unique features and can be distinguished from other diplectanids (including species of *Diplectanum*), presenting arguments for considering *Heteroplectanum* as a junior synonym of *Acleotrema*. Therefore, *Acleotrema kyphosi* Yamaguti, 1968, *Diplectanum girellae* (Johnston & Tiegs, 1922), *Heteroplectanum kyphosi* (Yamaguti, 1968) Oliver, 1987, *Acleotrema gibsoni* Young, 1970 and *Acleotrema heronensis* Young, 1970, are considered synonyms of *A. girellae*. This species differs from its congeners by having: a tubular MCO with the distal extremity recurved and bifid; and a sclerotised sac with radial musculature involving the proximal portion of the MCO. New geographical record for Palmyra Atoll.

###### Acleotrema parastromatei

Taxon classificationAnimaliaDactylogyrideaDiplectanidae

(Rakotofiringa, Oliver & Lambert, 1987) Domingues & Boeger, 2007

####### Type host.

*Parastromateus niger* (Bloch) (Carangidae).

####### Other host and localities.

*Parastromateus niger* from Madagascar (Rakotofiringa et al. 1987).

####### Current host.

*Kyphosus cinerascens* () (New host).

####### Site infection.

Gills.

####### Specimens deposited.

CHCM No. 541 (paratypes) (1 slide, 2 specimen).

####### Prevalence and mean intensity.

50 and 50 (n=2).

####### Remarks.

This species was originally described as *Heteroplectanum parastromatei* by Rakotofiringa et al. (1987). However, Domingues and Boeger (2007) considered the genus *Heteroplectanum* as a junior synonym of *Acleotrema*. Therefore, this species was transferred to *Acleotrema* as *A. parastromatei*. It has a haptor with two squamodiscs, each consisting of 25 to 27 rows of sclerotised pieces. *A. parastromatei* in *K. cinerasens* from off Palmyra Atoll represents both a new host and a new geographical record. Only one host was found parasitised.

##### Polyopisthocotylea Odhner, 1912

###### Heteraxinidae Unnithan, 1957

####### Cemocotylella elongata

Taxon classificationAnimaliaMazocraeideaHeteraxinidae

(Meserve, 1938) Price, 1962

######## Type host.

*Caranx melampygus* Cuvier (Carangidae).

######## Other host and localities.

*Caranx melampygus* from Secas Island, Panama (Meserve 1938). *Xurel melampygus* (=*Caranx melampygus*) (Cuvier and Valenciennes) from Secas Island, Panama (Price 1962). *Caranx latus* Agassiz from Chetumal, Quintana Roo, Mexico (Bravo–Hollis and Salgado–Maldonado 1983) (all Carangidae).

######## Current host.

*Caranx ignobilis* (Forsskål) (New host), *Caranx melampygus* and *Caranx papuensis* Alleyne and MacLeay (all Carangidae) (New host).

######## Site infection.

Gills.

######## Specimens deposited.

CHCM No. 536 (paratypes) (1 slide, 5 specimen).

######## Prevalence and mean intensity.

*Caranx ignobilis* 50 and 7±7 (n=4), *Caranx melampygus* 16,7 and 4 (n=6) and *Caranx papuensis* 40 and 7±7 (n=5)

######## Remarks.

Originally described as *Axine elongata* by Meserve (1938) from gills of *Caranx melampygus* (misidentified as *Xurel malampygus*). Price (1962) proposed the genus *Cemocotylella* to include *A. elongata*, changing the name of this species to *C. elongata*. *Axine elongata* (Meserve 1938) and *Heteraxine elongata* (Meserve 1938) Sproston 1946 are considered synonyms of *C. elongata*. This species is characterized by having an asymmetrical posterior haptor, four to five suckers on the short side and 24–25 on the long side, an unarmed genital atrium and MCO, and he absence of a vagina. The presence of *C. elongata* on *Caranx papuensis* from off Palmyra Atoll represents both a new host and a new geographical record.

###### Microcotylidae Taschenberg, 1879

####### Metamicrocotyla macracantha

Taxon classificationAnimaliaMazocraeideaMicrocotylidae

(Alexander, 1954) Koratha, 1955

######## Type host.

*Mugil cephalus* Linnaeus (Mugilidae).

######## Other host and localities.

*Mugil cephalus* from off Mexico (as *Microcotyle macracantha*) (Alexander 1954), the Gulf of California and Port Aransas, Texas from the same host (as *Metamicrocotyla macracantha*) (Koratha 1955), and *Mugil liza* from Brazil (as *Metamicrocotyla macracantha*) (Kohn et al. 1994). There are several reports of *Metamicrocotyla macracantha* from the USA on *M. cephalus* (Hargis 1956, Skinner 1975, 1978, Rawson 1976, Minchew 1977, Collins 1985), as well as from Australia (Young 1970), Mexico (Bravo–Hollis 1966, 1982, Juárez-Arroyo and Salgado-Maldonado 1989), Peru (Tantalean 1974), Puerto Rico (Garcia and Williams 1985), Chile (Oliva and Muñoz 1985, Bargiela 1987), and Venezuela (Conroy et al. 1985, 1986) from *Mugil curema* Valenciennes (all Mugilidae).

######## Current host.

*Crenimugil crenilabris* (Forsskål) (Mugilidae) (New host).

######## Site infection.

Gills.

######## Specimens deposited.

CHCM No. 552 (paratypes) (1 slide, 1 specimen).

######## Prevalence and mean intensity.

4,8 and 3±1 (n=42).

**Remarks**: *Metamicrocotyla macracantha* is characterized by having a haptor separated from the body proper by a peduncle and with 26–67 clamps disposed in 2 symmetrical lateral rows. The shape of haptor varies depending on state of contraction and number of clamps. Clamps of microcotylid type, similar in shape, somewhat variable in size; middle clamps are the largest, and those from anterior and posterior ends are the smallest. Testes rounded, normally 16 to 25 in a zigzag line occupying inter-caecal space. The presence of *M. macracantha* from the gills of *Crenimugil crenilabris* off Palmyra Atoll represents both a new host and a new geographical record.

###### Pterinotrematidae Bychowsky & Nagibina, 1959

####### Pseudopterinotrema albulae

Taxon classificationAnimaliaMazocraeideaPterinotrematidae

Yamaguti, 1966

######## Type host.

*Albula vulpes* Linnaeus (Albulidae).

######## Other host and localities.

Yamaguti (1966) recorded *P. albulae* from *A. vulpes* off Hawai’i. It has also been found on the same host by Palm and Bray (2014) off Hawai’i.

######## Current host.

*Albula glossodonta* (Forsskål) (Albulidae) (New host).

######## Site infection.

Gills.

######## Specimens deposited.

CHCM No. 535 (paratypes) (1 slide, 1 specimen), USNM No. 1459853 (voucher) (1 slide, 1 specimen).

######## Prevalence and mean intensity.

70,8 and 17±18 (n=24).

######## Remarks.

*Pseudopterinotrema albulae* presents an asymmetrical, fan-shaped haptor on a posterior extension of the body proper, with nine pedunculate clamps. The clamps have very distinct features (see Yamaguti, 1968 for a detailed description of each clamp). MCO plug-shaped, with two unequal sclerotised filaments at base; genital pore ventromedial. The presence of *P. albulae* from *A. glossodonta* off Palmyra Atoll represents both a new host and a new geographical record.

## Discussion

The species composition of monogeneans of the fishes from Palmyra Atoll is similar to that reported from other localities in the Indo-West Pacific and the Caribbean regions. These localities include the Great Barrier Reef (Australia), New Caledonia, Moorea (French Polynesia), South China Sea (Young 1968, Plaisance et al. 2004, Kritsky et al. 2009, Rehulkova et al. 2010) and Cuba (Zhukov 1976). Nine of the 16 species recorded in this study have been reported off Hawai’i (see Yamaguti 1968). This was not surprising, since Hawai’i is the closest location where monogeneans of marine fishes have been frequently examined. Some species recorded herein were previously considered endemic to Hawai’i; for example, *Pseudopterinotrema albulae*, *Benedenia hawaiiensis* and *Haliotrema minutospirale* (Palm and Bray 2014).

The absence of monogeneans from 26 of the 44 fish species examined was striking, even with relatively large sample sizes for some of those species (e.g. *Liza vaigiensis* n=54, *Valamugil engeli* n=63). Of those fishes that were infected, 14 of 18 species were parasitised by only one monogenean species. The low diversity of monogeneans found in our study is similar to that reported by Lafferty et al. (2008), who found only three species of monogeneans from five fish species captured on the forereef at Palmyra Atoll (n=11–25 individuals) and one monogenean species from a similar sample at the nearby Kiritimati Island. The species richness of monogeneans at Palmyra Atoll (16 monogenean species in 18 fish species) appears to be low compared with other localities, including the Hawaiian Islands, which are themselves remote and with some groups depauperate. Several host species from which monogeneans were absent in this study, have previous records of monogeneans. For example, *Pseudorhabdosynochus cupatus*, *P. melanesiensis*, *P. vagampullum* and *P. coioidesis* on *Epinephelus merra*; or *Pseudochauhanea sphyraenae* and *Vallisiopsis sphyraenae* on *Sphyraena barracuda*; or three *Haliotrema* species reported on *Stegastes nigricans* (Yamaguti 1968, Lo et al. 1998, Bu et al. 1999, Justine 2005, Hinsinger and Justine 2006).

The most likely hypothesis to account for the paucity of monogenean parasites at Palmyra Atoll is its geographical remoteness and small area. The Line Islands are isolated from other island groups in the Pacific and are also remote from the Austro-Malayan-Philippine region, the presumed centre of origin of Indo-West Pacific fishes and their parasites. Since there are also fewer species than those described from off Hawai´i, which is still further from the presumed centre of origin, we suggest the particularly small area of Palmyra Atoll contributes to the depauperate nature of its monogenean fauna. In fact, the low species richness of fishes from the Line Islands (Gosline 1971) is associated with the absence or scarcity of free-living species at Palmyra Atoll compared to other coral atolls in the Indo-West Pacific region (e.g. Adler 1992).

The presence of fish hosts, but often not their directly transmitted monogenean parasites, is consistent with the hypothesis that including a pelagic larval phase in marine animal life cycles is selectively advantageous because these small, morphologically and physiologically distinctive life history phases are incompatible with most of the parasites of juvenile and adult hosts (Strathmann et al. 2002).

This interspecific comparative study is consistent with the experimental studies (e.g., Grutter et al. 2011, 2017), that evaluate how parasites are transmitted to fish hosts after settlement from the pelagic region.

An additional explanation for the low number of monogenean species off Palmyra Atoll at local scale is related to the habitat from which almost all the fish examined were obtained: the lagoon flats. These flats are shallow and the daily temperature range is between 28.2 and 30.1°C (Koweek et al. 2014), perhaps offering an unsuitable environment for monogenean transmission or survival. For example, the negative effect of water temperature on the longevity and infection success of the oncomiracidia of *Neobenedenia* sp. infecting barramundi (*Lates calcarifer*) has been demonstrated (Hirasawa et al. 2010; Brazenor et al. 2013).

In conclusion, the number of species and individuals of monogeneans appear to be low in Palmyra lagoon-flat fishes. Filters acting at both local and biogeographical levels (*sensu* Holmes 1990, Combes 2001) seem to preclude the presence of a rich monogenean fauna. However, monogeneans were studied almost exclusively on fish from the lagoonal flats. The generally low infection prevalence is consistent with a more limited study of five fish species on coral reefs at Palmyra Atoll (Lafferty et al. 2008). Studies on the diversity of the coral reef fish fauna have found important differences in species number and composition between lagoonal flats, backreef and forereef zones (García-Sais 2010; Zhao et al. 2017). These ecological differences in reef zones could also contribute to differences in monogenean species richness and composition. Consequently, comparative studies of the monogenean fauna in different reef zones are needed to determine whether differences in monogenean diversity mirror differences in fish diversity. Palmyra Atoll has not had a permanent human population since WWII, and all fishing has been prohibited since it became a US National Wildlife Refuge in 2000. Consequently, the patterns and processes governing monogeneans diversity obtained in this relatively pristine environment could shed light on patterns of transmission prior to the removal of top predators by fishing, the situation found elsewhere.

## Supplementary Material

XML Treatment for Benedenia hawaiiensis

XML Treatment for Ancyrocephalus ornatus

XML Treatment for Euryhaliotrema annulocirrus

XML Treatment for Euryhaliotrema chrysotaeniae

XML Treatment for Euryhaliotrema grandis

XML Treatment for Haliotrema acanthuri

XML Treatment for Haliotrema aurigae

XML Treatment for Haliotrema dempsteri

XML Treatment for Haliotrema minutospirale

XML Treatment for Haliotrematoides patellacirrus

XML Treatment for Neohaliotrema bombini

XML Treatment for Acleotrema girellae

XML Treatment for Acleotrema parastromatei

XML Treatment for Cemocotylella elongata

XML Treatment for Metamicrocotyla macracantha

XML Treatment for Pseudopterinotrema albulae
